# DDS-type near-infrared light absorber enables deeper lesion treatment in laser photothermal therapy while avoiding damage to surrounding organs

**DOI:** 10.3389/fbioe.2024.1444107

**Published:** 2024-08-15

**Authors:** Masataka Takahashi, Jun Fujishiro, Shinsuke Nomura, Manabu Harada, Akinari Hinoki, Masashi Arake, Eiichi Ozeki, Isao Hara, Ayano Satoh, Takahisa Tainaka, Hiro-o Uchida, Yuji Morimoto

**Affiliations:** ^1^ Department of Pediatric Surgery, The University of Tokyo, Tokyo, Japan; ^2^ Department of Cell Engineering, National Center for Child Health and Development, Tokyo, Japan; ^3^ Department of Surgery, National Defense Medical College, Tokorozawa, Japan; ^4^ Department of Pediatric Surgery, Nagoya University Graduate School of Medicine, Nagoya, Aichi, Japan; ^5^ Department of Physiology, National Defense Medical College, Tokorozawa, Japan; ^6^ Technology Research Laboratory, Shimadzu Corporation, Kyoto, Japan; ^7^ Department of Applied Chemistry and Biotechnology, Faculty of Engineering, Okayama University, Okayama, Japan

**Keywords:** drug delivery system (DDS)-type near-infrared (NIR) absorbing agents, laser photothermal therapy, cell death, indocyanine green lactosome, orthotopic neuroblastoma tumor model

## Abstract

The efficacy of drug delivery system (DDS)-type near-infrared (NIR) absorbing agents in enhancing laser photothermal therapy is widely acknowledged. Despite the acknowledged efficacy, the therapeutic advantages of photothermal therapy using DDS-type NIR-absorbing agents over simple photothermal therapy without such agents have not been fully elucidated. This study was designed to investigate two primary objectives: firstly, the ability of DDS-type NIR-absorbing agents to induce cell death at greater depths within tumors, and secondly, their capacity to minimize collateral damage to adjacent healthy organs. To investigate these objectives, we employed a combination of indocyanine green lactosome—a DDS-type NIR-absorbing agent—and a precision-controlled laser hyperthermia system. An orthotopic neuroblastoma tumor model was used to closely simulate clinical conditions. The findings revealed that photothermal therapy using the DDS-type NIR-absorbing agent not only facilitates deeper penetration of cell death within tumors but also significantly mitigates thermal damage to surrounding healthy tissues, when compared to simple phototherapy without the agent. Furthermore, the combined treatment significantly prolonged the survival periods of the animals involved. This study is the first to analyze these therapeutic efficacies using quantitative data from an orthotopic tumor animal model and substantiated the potential of DDS-type NIR-absorbing agents to deepen the therapeutic impact of photothermal therapy while safeguarding vital organs, thereby enhancing overall treatment outcomes.

## 1 Introduction

In near-infrared laser photothermal therapy, a hyperthermic cancer treatment technique, a substance that absorbs near-infrared (NIR) light [e.g., indocyanine green (ICG)] is used in combination to heat the lesion with high efficiency, thereby improving treatment efficiency (Jung et al., 2018). There are two main routes for the administration of NIR-absorbing agents: one is direct injection into the tumor tissue and the other is intravenous administration. Although the former can concentrate the agent in the lesion, it is inferior to intravenous administration (Huang and Hainfeld, 2013; Li et al., 2016) due to several disadvantages: (1) it is difficult to distribute and diffuse the agent homogeneously throughout the solid tumor (Wolinsky et al., 2012), (2) it is difficult to avoid leakage into the surrounding healthy tissues, and (3) some organs are clinically difficult to access locally for direct injection (Kennedy et al., 2011). In addition, in recent years, drug delivery systems (DDSs) have been explored to selectively deliver NIR-absorbing agents to target lesions, and research and development of NIR-absorbing agents with DDS functions have been vigorously pursued. Many studies have shown that the use of intravenously administered DDS-type NIR-absorbing agents can increase the tumor temperature during light irradiation (5°C–20°C) (Yue et al., 2013; Yang et al., 2017) compared to that without such agents and enhance tumor growth inhibition.

However, the anti-tumor effects attributed to DDS-type NIR-absorbing agents might be replicable by simply increasing the light irradiation power to raise the tumor temperature sufficiently. This is because it is highly probable that the same anti-tumor effect can be achieved with or without the use of DDS-type NIR-absorbing agents as long as the light irradiation power is increased and the tumor temperature is raised by about 45°C (Hahl et al., 1990; Huang et al., 1991; Nomura et al., 2020).

We believe that one of the therapeutic advantages of using DDS-type NIR-absorbing agents is the ability to treat deeper lesions than when the agents are not used. Here, “critical depth” is defined as the depth at the limit of what can be treated with NIR light alone. There is a high probability that DDS-type NIR-absorbing agents that accumulate beyond the “critical depth” will absorb the NIR light with high efficiency, and the heat generated by this absorption (Liu et al., 2002; Yaseen et al., 2007) is likely to induce cancer cell death in areas beyond the “critical depth.” However, there have been few empirical studies using animal models; most studies have been simulations.

We believe that another therapeutic advantage of using DDS-type NIR-absorbing agents is the minimization of damage to surrounding healthy tissue (Schwartz et al., 2009). We speculated that DDS-type NIR-absorbing agents require less light energy to heat tumor tissue to a desired temperature compared to that required by methods that do not use these agents. This results in less thermal damage to adjacent healthy tissue. Studies on this issue have been conducted in humans and dogs (Schwartz et al., 2009; Rastinehad et al., 2019), and the possibility that thermal injury to adjacent healthy tissue may be less severe has been discussed but not quantitatively evaluated. In most studies, the photothermal effects of newly developed DDS-type NIR-absorbing agents have been investigated by using subcutaneous (intradermal) tumor model mice, and thermal damage to healthy tissue surrounding the tumor was only mentioned in a few reports (Yang et al., 2017). In mice with subcutaneous (intradermal) tumors, the surrounding healthy tissue (interstitial tissue such as fat and muscle) is very thin, making it difficult to evaluate pathological changes due to thermal damage. In addition, subcutaneous (intradermal) tumor models are unlikely to reflect clinical pathology.

As mentioned above, many studies have not adequately demonstrated the therapeutic advantages of using DDS-type NIR-absorbing agents. Therefore, we investigated the following two issues to demonstrate the utility of DDS-type NIR-absorbing agents.

1. Can the use of DDS-type NIR-absorbing agents increase the tumor depth at which cell death can be induced?
2. Can the use of DDS-type NIR-absorbing agents reduce the damage to organs surrounding the tumor?

To investigate these objectives, we used a DDS-type NIR-absorbing agent, a laser photothermal therapy system that can maintain and control lesion temperature, and an orthotopic animal model of cancer that mirrors the clinical condition of the disease.

In this study, we used neuroblastoma, which originates in the adrenal glands or the sympathetic ganglions close to vital organs (kidney, spinal canal, aorta), as an orthotopic model. Neuroblastoma invades the spinal canal in 5%–15% of cases, resulting in an oncologic emergency (Sorrentino et al., 2022). In such cases, emergency radiation therapy or decompression surgery is typically performed; however, the efficacy of these treatments is limited. There is currently no established lesion-selective therapy, and there is a need for methods that preserve surrounding organs. This makes the neuroblastoma model ideal for assessing the efficacy of photothermal therapy using DDS-type NIR-absorbing agents.

## 2 Materials and methods

### 2.1 DDS-type NIR-absorbing agent

#### 2.1.1 ICG lactosome

**FIGURE 1:**
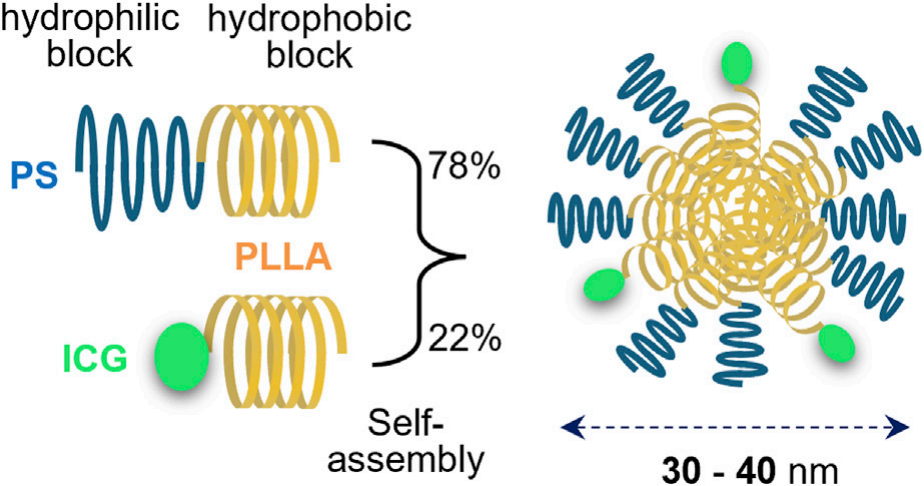
Structure of indocyanine green lactosome (ICG-lac). ICG-lac is a polymeric micelle-loaded ICG. It is synthesized by self-assembly of block copolymers polysarcosine–poly L-lactic acid (PS-PLLA) and ICG–poly L-lactic acid (ICG-PLLA) in an aqueous solution and has a particle size of 30–40 nm.

Indocyanine green lactosome (ICG-lac) was used as a DDS-type NIR-absorbing agent in this study. ICG-lac is a polymeric micelle loaded with ICG molecules (Makino et al., 2007) (Figure 1), and it accumulates in tumors with high efficiency due to its enhanced permeability and retention (EPR) effect (Matsumura and Maeda, 1986). The structure is formed by self-assembly of amphiphilic block copolymers (particle sizes of 30–40 nm). The hydrophobic block consists of polylactic acid, while the hydrophilic block consists of 78% polysarcosine and 22% ICG. Both polylactic acid and polysarcosine are composed of FDA-approved biodegradable polypeptides, and both have minimal toxicity, suggesting that they can be used safely in humans. The ICG molecule strongly absorbs NIR light in the 800 nm wavelength range, enabling photothermal treatment of tumors (Tsujimoto et al., 2014; Tsuda et al., 2017). In addition, because ICG molecules are also fluorescent, lesions can be easily tracked and observed in real time by fluorescence imaging (Makino et al., 2007; Makino et al., 2009), allowing precise control of the treatment area.

#### 2.1.2 Administration of ICG lactosomes

Dried ICG lactosomes were dissolved in pure water and administered intravenously at a dosage of 8.8 mg/kg *via* the posterior orbital sinus using a 29 G needle. The molar concentration per blood is 281 μM, assuming the mouse weighs 20 g and the blood volume is 1/12 of the body weight.

### 2.2 Evaluation of therapeutic efficacy using an orthotopic tumor animal model

#### 2.2.1 Preparation of orthotopic tumor animal models

##### 2.2.1.1 Methods for establishing and culturing gene-transfected cells

The luminescent gene nano-lantern was transfected into mouse-derived neuroblastoma cells (C1300, RCB Cat# RCB0283, RRID:CVCL 4343) via a retrovirus (Saito et al., 2012), and the stable expression cell line nano-lantern C1300 (C1300-NL) was established. Cells were cultured in RPMI medium (Sigma-Aldrich, Cat #R8758) supplemented with 10% FBS (Thermo Fisher Scientific, Cat # 16000044), 100 U/mL penicillin, 100 μg/mL streptomycin, and 0.25 μg/mL amphotericin B (Antibiotic-Antimycotic, Thermo Fisher Scientific, Cat # 15240062). Cells were cultured in an incubator at 37°C with 5% CO_2_ and balanced air.

##### 2.2.1.2 Establishment of orthotopic neuroblastoma mouse models

All of the following animal procedures were performed in accordance with the guidelines approved by the National Defense Medical College Animal Care and Use Committee (Permit number: 19009). Six-week-old female A/J mice (A/JJmsSlc, Japan SLC, RRID: MGI:2160468) were used.

A/J mice were intraperitoneally injected with a mixture of anesthetics: medetomidine (0.3 mg/kg, Meiji Animal Health, Japan), midazolam (4.0 mg/kg, Sandoz), and butorphanol (5.0 mg/kg, Meiji Animal Health, Japan). Subsequently, a C1300-NL cell suspension, composed of 2 × 10E6 C1300-NL cells in a 2:1 mixture of HBSS (Thermo Fisher Scientific, Cat # 14170112) and Matrigel (Corning Cat # 354234), was injected into the left adrenal gland (20 µL). The transplantation procedure was performed according to previous publications (Fuchs et al., 2009; Chiu et al., 2014). Briefly, with the mouse in the supine position, a longitudinal incision was made just above the left adrenal gland, exposing the retroperitoneal cavity around the left kidney. The surrounding tissues of the left adrenal gland, located cephalically medial to the upper pole of the left kidney, were carefully dissected and maneuvered into a visible position. The left adrenal gland was then grasped with the left hand to prevent movement. The cell suspension was then injected into the left adrenal gland by direct puncture with a 29 G needle. The mice had a 79% (90 of 114) chance of developing tumors.

#### 2.2.2 Evaluation of tumors

##### 2.2.2.1 *In vivo* imaging for luminescence to confirm tumor localization and fluorescence to confirm ICG lactosome localization

When coelenterazine, a luminescent substrate, is incorporated into C1300-NL, coelenterazine is degraded by the nano-lantern and emits high-intensity luminescence. This phenomenon can be used to visualize the localization of tumors formed by C1300-NL. In the experiment, 100 µL of 2.5 mg/mL coelenterazine h (FUJIFILM Wako Pure Chemical Corporation, Japan, Cat #031-22993) solution was injected intravenously through the posterior orbital sinus into an orthotopic mouse model. Using an *in vivo* imaging system (IVIS, PerkinElmer), coelenterazine luminescence images and bright-field mode images were captured and superimposed to visualize tumor distribution.

ICG fluorescence imaging (excitation wavelength 780 nm/fluorescence wavelength 845 nm, 1 s exposure) was also performed to visualize ICG-lac uptake within the tumor using IVIS.

##### 2.2.2.2 *Ex vivo* fluorescence imaging for excised tumor tissues

Excised specimens were sectioned at the center of the largest tumor, embedded in OCT compound (Sakura Finetek, Japan, Cat #4583), and quickly frozen in liquid nitrogen. The specimens were then immediately transferred to a cryostat (−20°C), sectioned at 5 μm in thickness, and mounted on glass slides. Immediately after, ICG fluorescence distribution was observed using a fluorescence microscope (BZ-X800, Keyence, Japan) with a filter block (Cat#49030-UF1-BLA: excitation 750–800 nm, dichroic mirror 810 nm, fluorescence 817.5–872.5 nm. Chroma). The obtained images were compiled and processed using an image analysis application (BZ-H4A, Keyence, Japan). To evaluate the fluorescence intensity, the fluorescence intensity of the tumor and normal tissue (kidney) within a defined region of interest (ROI) was compared to the background fluorescence and the difference was calculated. The ratio of this difference was calculated using the following formula ([tumor fluorescence ROI - background fluorescence ROI]/[kidney fluorescence ROI - background fluorescence ROI]).

##### 2.2.2.3 *In vivo* ultrasound imaging

A high-resolution small animal ultrasound imaging system (VEVO 770, Visual Sonics, Canada) was used for imaging to measure tumor size and determine characteristics. An ultrasound probe (RMV704, center frequency of 40 MHz, spatial resolution of 80 μm, Visual Sonics) was placed on the dorsal skin of the mouse.

##### 2.2.2.4 Histopathological examination

The excised tumors and organs were fixed in 10% formaldehyde, embedded in paraffin, sectioned thinly, and stained with hematoxylin-eosin stain (H-E stain). After capturing images using the BZ-800X microscope, the images were concatenated and processed using the BZ-H4A analysis application. Subsequently, the treatment depth was accurately measured with the BZ-H4M application.

#### 2.2.3 Mouse survival assessment

Tumor size was measured using the VEVO 770 ultrasound imaging system on day 7 after transplantation of C1300-NL into mice. Tumor luminescence was confirmed using the IVIS imaging system only when the tumor size was less than 3 mm. This was done to accurately verify that what had been identified as a tumor by ultrasound imaging was indeed a tumor. The total number of mice transplanted with C1300-NL cells was 30, and tumors were confirmed in 21 of them.

Four experimental groups including (1) a control group (no ICG-lac administration and no NIR irradiation), 2) ICG-lac administration only group, 3) NIR irradiation only group, and 4) ICG-lac administration + NIR irradiation group were established and mice were randomly assigned to each group starting with the largest tumor size. In experimental groups (2) and (4), ICG-lac was administered on day 7 after cell transplantation.

For mice in all experimental groups, the retroperitoneum was incised 10 days after cell transplantation to visually confirm adrenal tumors. At that time, mice that died due to diaphragmatic injury and mice in which the tumor were damaged were excluded. After confirmation of the adrenal tumor, the wound was closed after irradiation in experimental groups (3) and (4) but not in experimental groups (1) and (2). As a result, the numbers of mice in groups (1), (2), (3) and (4) were 5, 4, 3, and 4, respectively, and those mice were used for subsequent analysis. For photothermal heating by NIR irradiation, the temperature setting to maintain heating of the tumor surface was 45°C. After the start of NIR irradiation, the irradiation was continued for another 300 s from the time when the temperature reached the set temperature. The animals were then observed under normal housing conditions until death.

### 2.3 Laser photothermal system

A laser photothermal system developed by the authors (Nomura et al., 2017; Nomura et al., 2020) was used for photothermal heating. The system is a constant-temperature NIR irradiation system that continuously heats the irradiated object at a predetermined constant temperature. The principle of the system is that the surface temperature of the irradiated object is monitored with a thermal imaging camera (FSV-210L, Apiste, Japan) and the output of NIR light is controlled on the basis of the temperature information to keep the lesion at a predetermined temperature (temperature resolution: 0.1°C) during light irradiation. The system is equipped with an 808 nm laser diode (model FC-W-808, Changchun New Industries Optoelectronics Technology, China). When irradiating mice, the irradiation fiber probe was adjusted to be positioned directly above the target (exposed adrenal tumor). The size of the irradiation spot was 0.5–1.5 cm in diameter and was adjusted according to the size of the lesion.

### 2.4 Measurement of internal tumor temperature

Needle thermocouples were used to measure the internal temperature of the tumor during photothermal heating.

Only animals with confirmed tumors larger than 5 mm were included in the experiment and were treated with ICG-lac. On the third day after ICG-lac administration, the dorsal skin was incised to expose the left adrenal tumor. A T-type (copper-constantan) needle thermocouple (OD = 0.2 mm) (HYP0-33-1-T-G-60-SMPW-M, OMEGA Engineering, Japan) was then inserted from the outside of the tumor and advanced into the tumor so that the long axis of the needle was parallel to the light-illuminated surface (Supplementary Figure S1A). Two to three thermocouples were used depending on the size of the tumor. NIR irradiation was then applied, and the tumor surface temperature was measured by thermography (FSV-210L) and the internal tumor temperature was measured by the thermocouples (sampling interval: 1 s) (Supplementary Figure S1B). After NIR irradiation, the distance from the light-irradiated surface level of the tumor to the tip of the needle thermocouple was measured using the VEVO770 ultrasound imaging system, and the distance was defined as “depth” (Supplementary Figure S1C). The relationship between depth and temperature inside the tumor during NIR irradiation was then investigated.

Two experimental groups were established: (a) an ICG-lac-treated group and (b) a control group (no ICG-lac treatment). During the experimental manipulations, mice in which the needle thermocouple was dislodged, mice in which the location of the thermocouple was not clear on ultrasound examination, and mice that died were excluded from subsequent analysis. The resulting numbers for analyses were n = 8 for (a) and n = 10 for (b).

### 2.5 Statistical analysis

GraphPad Prism for MAC (ver. 8.3.0, GraphPad Software) was used for statistical analysis. Survival curves were plotted using the Kaplan-Meier method, and log-rank tests were used for between-group comparisons. Generalized regression analysis was performed for the bivariate relationship between temperature and depth. The Fisher’s test was used to compare the presence or absence of renal injury between the two groups. A *p*-value of less than 0.05 was considered significant.

## 3 Results

### 3.1 Excellent tumor-accumulating properties of ICG lactosomes

#### 3.1.1 Consistent accumulation of ICG lactosomes in tumors

We investigated the selective tumor accumulation and localization of ICG lactosomes using an orthotopic neuroblastoma mouse model. Mice were intravenously administered ICG lactosomes after implanting C1300 cells expressing the luminescent nano-lantern gene into their adrenal glands.

**FIGURE 2:**
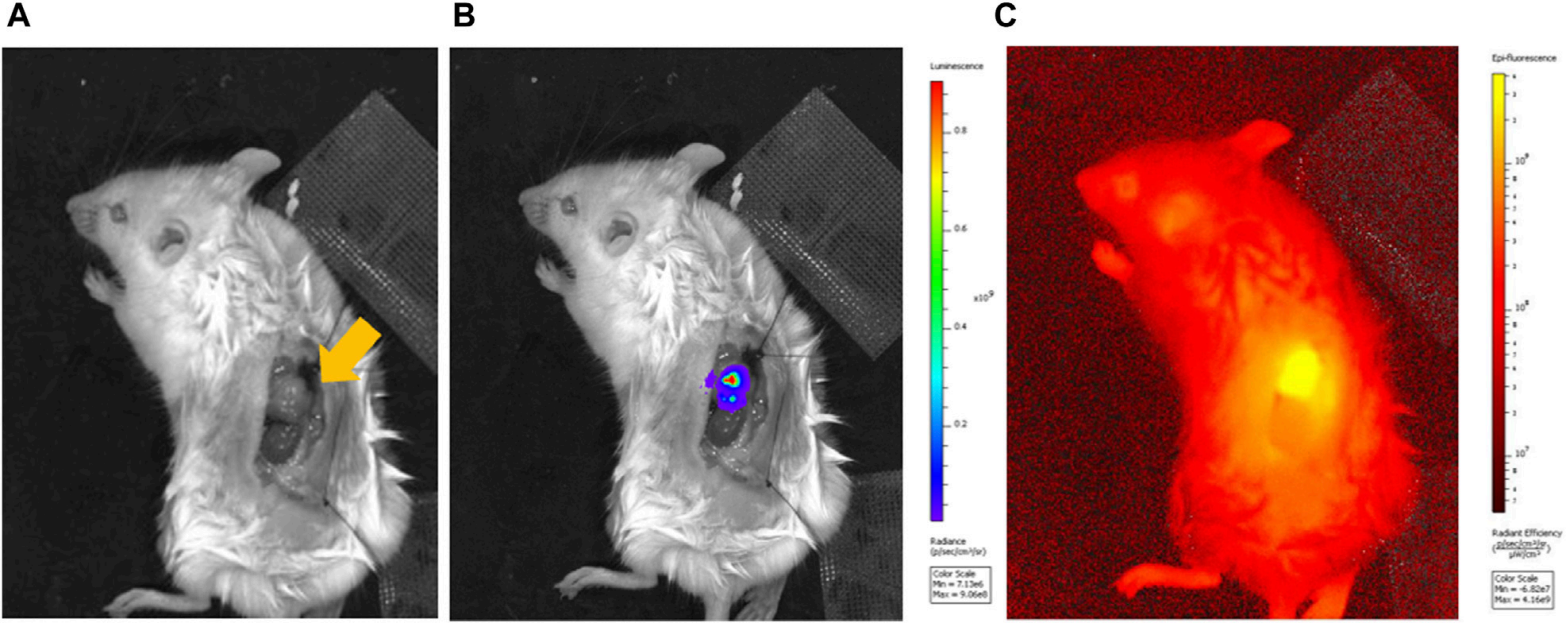
Luminescence imaging of a mouse model of orthotopic neuroblastoma of the left adrenal gland and fluorescence imaging of ICG-lac accumulated in the tumor. **(A)**, Bright field image of adrenal neuroblastoma (arrow). **(B)**, Luminescence image after administration of a luminescent substrate (coelenterazine h). **(C)**, Fluorescence image originating from ICG-lac (day 3 after ICG-lac administration).

*In vivo* imaging showed clear luminescence at the tumor sites within the adrenal glands (Figure 2). ICG fluorescence imaging further confirmed selective accumulation at these locations.

#### 3.1.2 Extensive accumulation of ICG lactosomes in the tumor

With the prediction that greater accumulation would enhance the thermal effect of NIR irradiation (Makino et al., 2012; Tsuda et al., 2017), we measured the peak accumulation times of ICG lactosomes.

**FIGURE 3:**
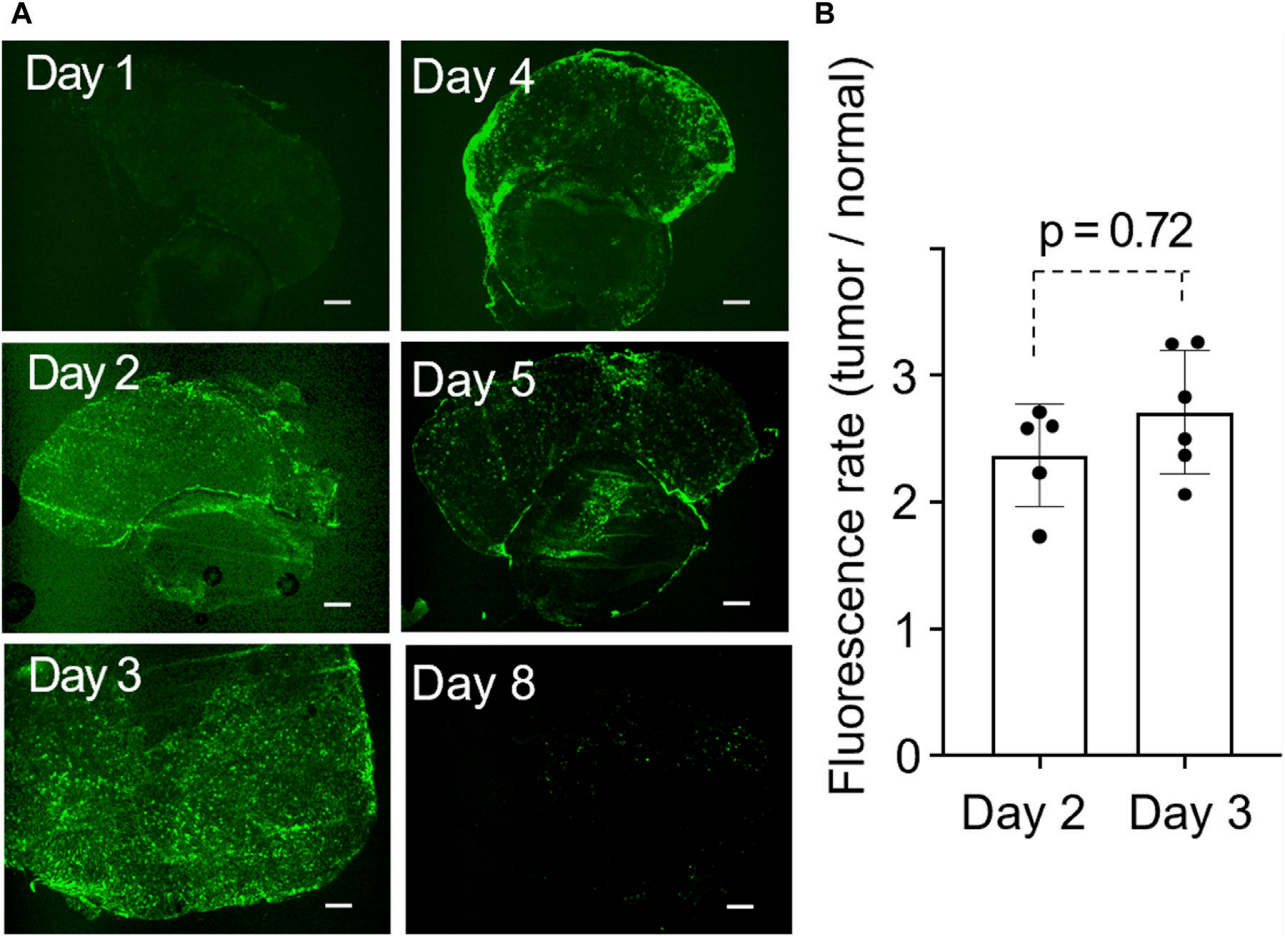
**(A)**, Time-dependent change in the distribution of ICG-lac inside the tumor. The tumors shown in the six panels were obtained from distinct individuals. Scale bar = 300 µm. **(B)**, Quantitative fluorescent rates in tumors on day 2 and day 3 after the administration of ICG-lac.

Fluorescence microscopy revealed a homogeneous distribution inside the tumor for the first 3 days post-administration. From the fourth day, the lactosomes predominantly accumulated at the tumor margins, with a noticeable decrease by the eighth day (Figure 3A). Our quantitative analysis showed no significant difference in the fluorescence intensity on the second and third days post-administration, suggesting that these days were optimal times for photothermal treatment (Figure 3B).

### 3.2 Increased treatment depth with ICG lactosomes

#### 3.2.1 Maximum treatment depth of 9 mm with the use of ICG-lac

We investigated whether photothermal therapy with ICG-lac had a therapeutic effect on deeper lesions compared to mere photothermal therapy. The depth was evaluated by maintaining a constant temperature of 45°C during NIR irradiation.

**FIGURE 4:**
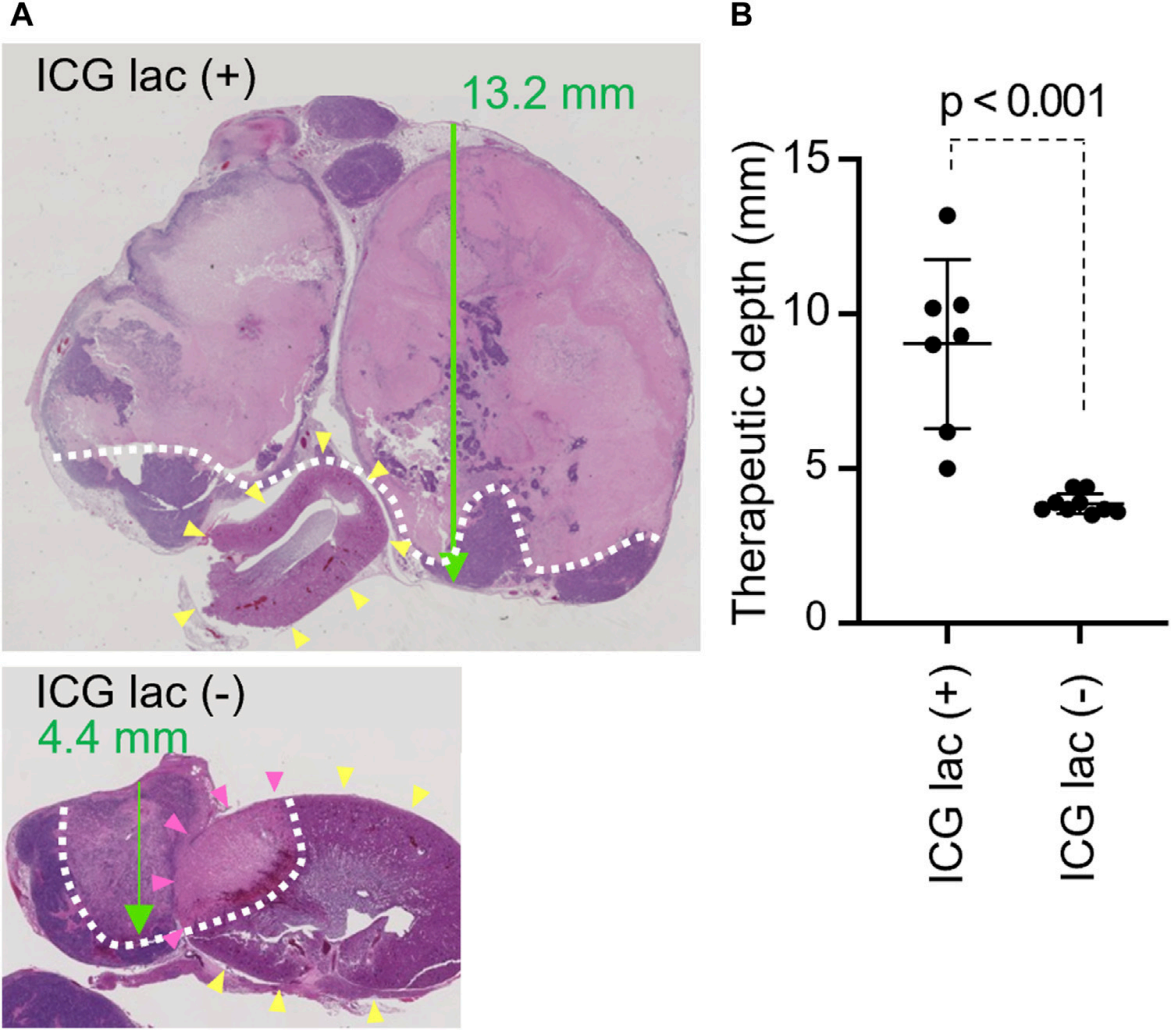
Therapeutic depth after photothermal therapy with/without ICG-lac. **(A)**, Pathological findings of each case (H and E staining). Green arrow: penetration depth. Yellow arrowheads: kidney. White dotted line: boundary of necrotic area. Tumor coagulative necrosis is seen with or without ICG-lac, but treatment depth is greater with ICG-lac. In the case without ICG-lac, coagulative necrosis is seen in parts of the kidney (pink arrowheads). **(B)**, Comparison of the therapeutic depths for photothermal therapy with ICG-lac (n = 7) and photothermal therapy (45°C) without ICG-lac (n = 9).

Histopathological analysis revealed that the maximum necrosis depth was significantly greater in the ICG-lac group (9.0 ± 2.7 mm) than in the non-ICG-lac group (3.9 ± 0.3 mm; *p* < 0.0001) (Figure 4B). This indicates that the depth of NIR treatment in the ICG-lac group was more than twice that of simple NIR treatment when the tumor heat maintenance temperature was set at 45°C.

#### 3.2.2 Increase in tumor internal temperature due to the use of ICG lactosomes

In order to assess the thermal state of the tumor during NIR irradiation, the temperature within the tumor was quantified using needle thermocouples. The temperature was maintained at 45°C during NIR irradiation, and the maximum temperature during photothermal heating was measured at each distance from the tumor surface to the depth of the tumor.

**FIGURE 5:**
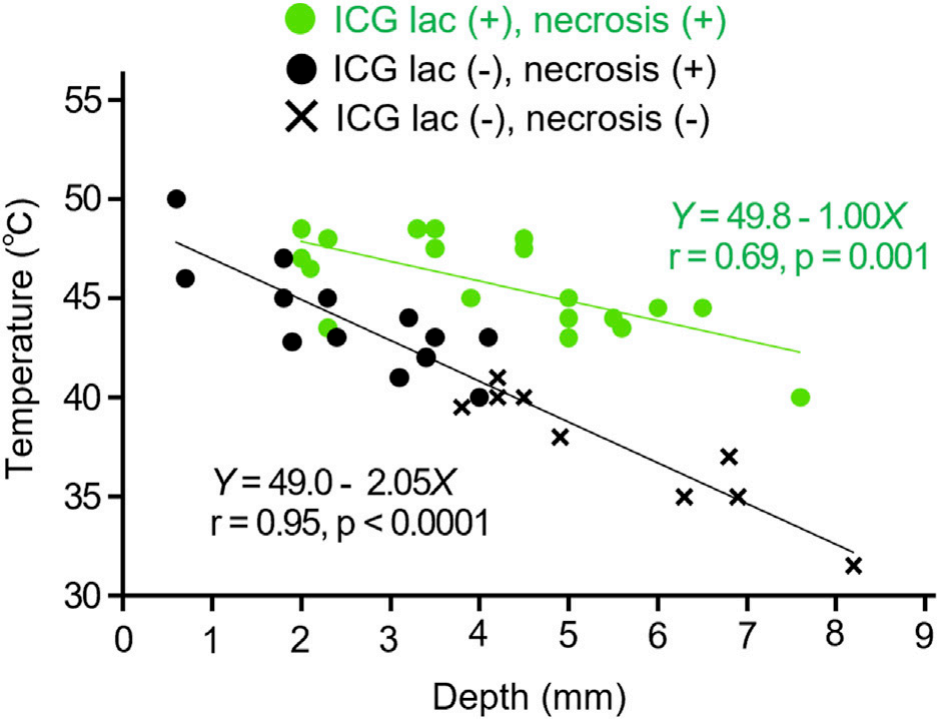
Relation between depth and temperature inside tumors during NIR irradiation. Comparison in cases of ICG-lac (+) (n = 19) and ICG-lac (−) (n = 22). Data with necrosis seen in the tumor tissue are plotted as ● and data without necrosis are plotted as ×.

As a result, a negative correlation between tumor depth and temperature was confirmed as shown in Eqations 1, 2 below (see also Figure 5).

$$\text{Internal temperature }\left({}^{\circ}C\right)\text{ of ICG}-\text{lac treated group}=49.8\;-\;1.0\times \text{depth }\left(\text{mm}\right)$$ (1)

$$\text{Internal temperature }\left({}^{\circ}C\right)\text{ of the group not treated with ICG}-\text{lac}=49.0\;-\;2.1\times \text{depth }\left(\text{mm}\right)$$ (2)

The coefficients of temperature attenuation were 1.0 for the ICG-lac group (Equation 1 and 2.1 for the non-ICG-lac group (Equation 2). The former value was approximately half that of the latter value. In other words, the use of ICG-lac made deeper heating possible. This corroborates the results presented in Section 3.2.1 showing that the necrosis depth in the ICG-lac group was greater than that in the non-ICG-lac group.

### 3.3 Photothermal therapy with ICG-lac induces coagulative necrosis of tumors without causing damage to surrounding organs

To evaluate whether the use of ICG-lac could mitigate the injury to healthy organs (kidneys) surrounding the tumor caused by photothermal heating, the tumor and kidneys were excised at 3 days after the NIR irradiation and observed histopathologically.

Under constant irradiation at 45°C, tumors exhibited coagulative necrosis regardless of ICG-lac treatment. In contrast, NIR light alone without ICG-lac led to coagulative necrosis in the kidneys of some animals (Figure 4A). To semi-quantitatively assess the injury, the presence or absence of coagulative necrosis in the renal parenchyma was examined histopathologically, comparing ICG-lac-treated animals (n = 8) and non-ICG-lac-treated animals (n = 10). As shown in Table 1, there were no cases of renal injury in the ICG-lac-treated group, whereas 20% (2 out of 10) of the non-ICG-lac-treated animals had renal injury.

**TABLE 1:** Renal injury caused by photothermal therapy to tumors: Differences with and without ICG-lac administration.

	Renal injury	
	(+)	(−)
ICG lac (+)	0	8
ICG lac (−)	2	8

Renal injury was defined as (+) when coagulative necrosis was evident in the kidney. In the ICG-lac (−) group, 20% of the animals showed renal injury, but none of the animals in the ICG-lac (+) group showed renal injury (*p* = 0.26, Fisher’s test).

### 3.4 Photothermal therapy with ICG-lac prolongs the survival period

**FIGURE 6:**
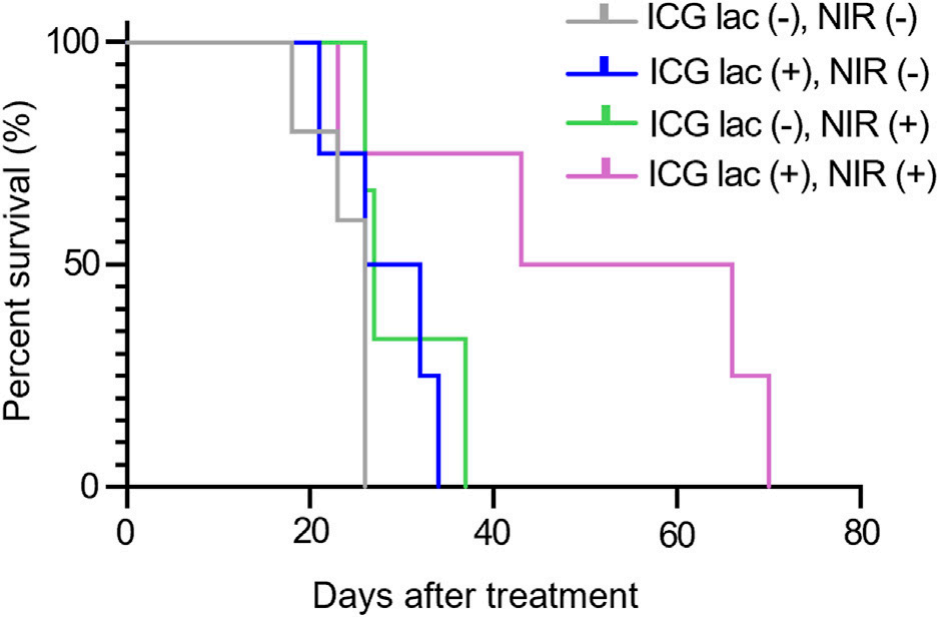
Survival rate after photothermal therapy in mice. Survival period was significantly prolonged in the group that received ICG-lac followed by photothermal therapy compared to the survival periods in the other three groups (*p* = 0.036, Log rank test).

As shown in Figure 6, the median survival period for the group treated with ICG-lac followed by NIR irradiation (ICG lac + NIR+) was 54.5 days. This duration was significantly longer than the median survival periods of 26 days (ICG lac- NIR-), 29 days (ICG lac + NIR-), and 27 days (ICG lac- NIR+) (*p* = 0.036, log-rank test). Note that all mice at the time of death were judged to have died of tumors, as the tumors were enlarged in all cases.

## 4 Discussion

In this study, laser photothermal therapy using ICG-lac was performed on an orthotopic model of neuroblastoma to clarify the therapeutic advantages of DDS-type NIR-absorbing agents. The results showed that the use of DDS-type NIR-absorbing agents increased the depth of the tumor at which cell death could be induced while also simultaneously reducing the injury to surrounding healthy organs. These effects contributed to prolonging the survival period of the tumor animals.

### 4.1 Expanding treatment depth with DDS-type NIR-absorbing agents

The combination of ICG-lac and laser photothermal treatment successfully reduced the tumor to an average depth of approximately 9 mm, reaching up to 12 mm at maximum. This result indicates that the heating range expanded by approximately 5 mm, achieving a depth of 9 mm compared to just 4 mm with photothermal treatment alone. One of the reasons for this is that the accumulation of a DDS-type NIR-absorbing agent in the tumor leads to more efficient heat production inside the tumor than in a tumor without NIR-absorbing agent accumulation (Alamdari et al., 2022).

In a previous study, *ex vivo* experiments using ICG and 808 nm NIR light on excised human pancreatic cancer specimens showed that the temperature inside the tumor was almost 4°C higher than the temperature of the tumor surface (Maziukiewicz et al., 2019). Also in experiments using other NIR-absorbing agents (gold nanorods), the temperature inside the tumor (about 3 mm from the surface) was higher than the temperature at the tumor surface with the temperature differences reaching 5°C–10°C (Mooney et al., 2017). Simulation studies (805 nm NIR irradiation of a phantom with ICG accumulation) also showed that the temperature about 2 mm deeper than the absorber surface was higher than the surface temperature (Liu et al., 2002).

When NIR-absorbing agents accumulate in tumor tissue, light energy can be converted into heat with high efficiency. This means that even low-intensity light, which typically decreases in intensity with depth, can effectively heat tumor tissue when an NIR-absorbing agent is present. This suggests that it is possible to treat tumors in deeper regions.

### 4.2 Possibility of treatment without damage by using DDS-type NIR-absorbing agents

Tumor tissue is more sensitive than healthy tissue to heat, and apoptosis is induced in general tumor cells when the temperature reaches 42°C–43°C. On the other hand, healthy cells (non-tumor cells) can tolerate this level of heat, and photothermal heat of about 45°C is said to have little effect on surrounding healthy tissue (Dewhirst et al., 2003). However, temperatures above 50°C have been shown to induce necrosis in tumors and surrounding tissues (Pérez-Hernández, 2019).

When irradiated with the same light intensity, the temperature at the site with accumulated NIR-absorbing agents rises more than at sites without these agents, allowing for more efficient induction of cell injury (Zhou et al., 2015). In other words, if the light intensity is such that cell injury begins only slightly at the site of accumulation of the NIR-absorbing agent, no cell injury occurs at the site of non-accumulation of the agent. This phenomenon is believed to have been observed in this study. Specifically, when ICG-lac was used, it accumulated solely in the tumor tissue, where it readily absorbed light. This accumulation is thought to have enabled tumor regression even with light intensities that did not cause thermal injury to healthy tissue. On the other hand, when ICG-lac was not used, a higher light intensity was required to reach the set temperature value for the tumor temperature, which is assumed to have resulted in damage to the adjacent healthy tissue.

In this study, the holding temperature during photothermal heating was set at a constant 45°C. However, it was initially expected that the anti-tumor effect would be greater if the temperature setting was increased. We conducted a preliminary experiment in which the tumor temperature was maintained at 50°C; however, no difference in the deep thermal effect was observed compared to that in the 45°C setting. A previous study on laser photothermal therapy using an intra-tumor temperature control system also showed that the therapeutic effect at a setting of approximately 43°C was not different from that at higher temperature settings, but rather that damage to surrounding healthy tissue was problematic (Zhu et al., 2016).

It is recommended that the heating temperature during photothermal therapy be limited to the necessary minimum to enhance safety for the living body (Wei et al., 2020).

### 4.3 Excellent tumor accumulation of ICG lactosome

In order to achieve optimal anti-tumor effects while minimizing damage to surrounding healthy tissue, DDS-type NIR-absorbing agents must be capable of selectively accumulating in tumors with minimal accumulation in the surrounding healthy tissue. The ICG-lac used in this study is a DDS-type NIR-absorbing agent that fully meets these criteria. ICG-lac demonstrates a high capacity to evade the reticuloendothelial system, thereby maintaining stability within the bloodstream. Consequently, ICG-lac exhibits gradual accumulation in tumors following administration due to the enhanced permeability and retention (EPR) effect (Makino et al., 2009). Following administration of ICG-lac to tumor-bearing mice, while some accumulation was observed in the liver and spleen, the accumulation in the tumors increased over time, reaching a tumor/background ratio of approximately 5 after 48 h (Makino et al., 2012). Surprisingly, even in a mouse orthotopic liver tumor model, the tumor/liver ratio increased over time, reaching more than twice the initial value between 30 and 50 h after administration (Makino et al., 2009).

### 4.4 Limitations

Firstly, it is important to clarify that the results of this study are not necessarily applicable to all DDS-type NIR-absorbing agents. In other words, among the many agents that have been developed, only ICG-lac was used in this study, which limits the ability to generalize the observed events. However, DDS-type NIR-absorbing agents with tumor-selective accumulation are likely to enable deeper treatment and reduce damage to surrounding organs to varying degrees compared to those without such selectivity. This is because, as mentioned above, the tumor-selective accumulation of NIR-absorbing agents is likely to induce more efficient heat production inside the tumor than in surrounding healthy organs (Alamdari et al., 2022).

In addition, the tumor model used in this study is a homogeneous tumor based on a single cell type. It is important to note that actual tumors are heterogeneous, and even tumors with the same histology can show varied drug accumulation (Kalisvaart et al., 2022), and the results of this study might therefore not be universally applicable. Hence, examining the distribution of ICG-lac within tumors using a tissue-based xenograft model is required (Yakavets et al., 2019). Additionally, the optimal timing between drug administration and light irradiation, which is crucial for effective treatment, may vary for different tumor types (Polom et al., 2011), and that also needs to be considered.

## 5 Conclusion

The use of DDS-type NIR-absorbing agents in laser photothermal therapy has been shown to (1) enhance the depth of treatment and to (2) permit the selective targeting of lesions without causing damage to surrounding organs. Consequently, survival period was prolonged, indicating that the aforementioned advantages are crucial for enhancing the efficacy of treatment.

## Data Availability

The original contributions presented in the study are included in the article/Supplementary Material, further inquiries can be directed to the corresponding authors.
