# Supplementary figures and images for: DDS-type near-infrared light absorber enables deeper lesion treatment in laser photothermal therapy while avoiding damage to surrounding organs
Source: Front Bioeng Biotechnol. 2024 Aug 15;12:1444107. doi: 10.3389/fbioe.2024.1444107 (PMC11357940; doi:10.3389/fbioe.2024.1444107)

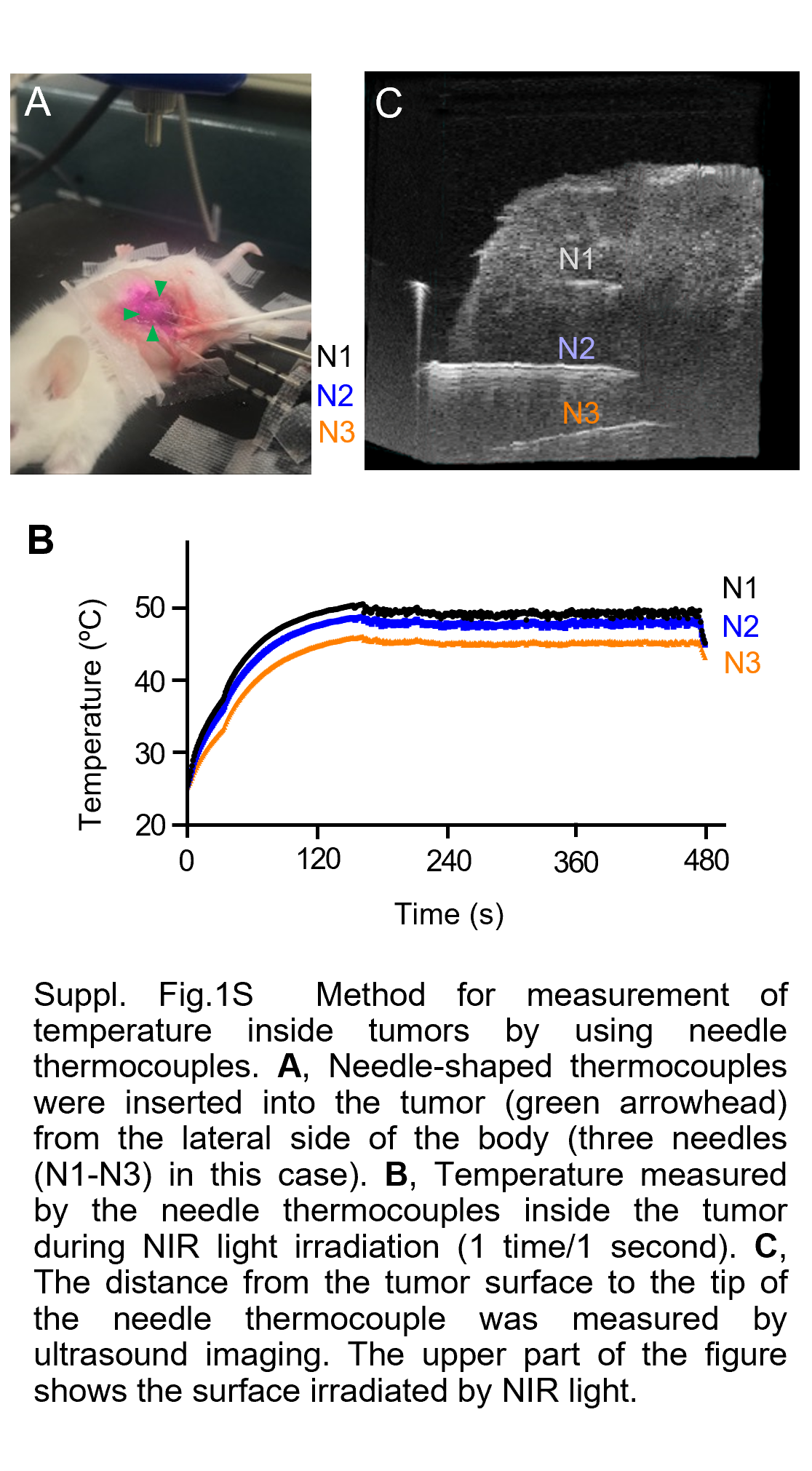

Supplement: Supplementary file 1 [file Image1.TIF]
